# The Association between Serum Uric Acid and Residual ****β****-Cell Function in Type 2 Diabetes

**DOI:** 10.1155/2014/709691

**Published:** 2014-05-26

**Authors:** Wei Tang, Qi Fu, Qingqing Zhang, Min Sun, Yuan Gao, Xuan Liu, Li Qian, Shan Shan, Tao Yang

**Affiliations:** ^1^Department of Endocrinology and Metabolism, The Affiliated Jiangyin Hospital of Southeast University Medical College, Jiangyin, Jiangsu 214400, China; ^2^Department of Endocrinology, The First Affiliated Hospital of Nanjing Medical University, Nanjing, Jiangsu 210029, China

## Abstract

The aim of this study was to investigate the relationship of serum uric acid (sUA) with residual **β**-cell function in type 2 diabetes. Oral glucose tolerance tests (OGTT) were performed on 1021 type 2 diabetes patients. The ratio of area under curve of insulin to glucose during 0 to 30 min and 0 to 120 min of the OGTT was calculated as indices of insulin secretion function. The products of insulin secretion indices multiplied by Matsuda insulin sensitivity index were used as disposition indices. After correlation and multiple linear regression analysis, sUA was significantly associated with insulin secretion and disposition indices in male, female, and total groups adjusted for confounding factors (including metabolic indicators like sex, age, course of the disease, blood glucose, blood pressure, serum lipids, and so on). Superficially steeper time-dependent decline of insulin secretion function was found in patients with sUA above the median than those below it. In conclusion, our results suggest an independent positive association between sUA and residual **β**-cell function in type 2 diabetes. Patients with higher sUA have greater insulin secretion ability than those with lower sUA at the early stage of disease, but their residual **β**-cell function seems to decay more rapidly.

## 1. Introduction

Lots of studies found increased serum uric acid (sUA) levels in subjects with metabolic syndrome (MetS) or cardiovascular disease, and sUA is associated with several components of MetS, including dyslipidemia, hypertension, impaired glucose metabolism, and obesity [1–3]. Some recent studies have already highlighted the connection between sUA and glucose homeostasis. For example, the Rotterdam Study showed that the subjects with higher levels of sUA were at higher risk of type 2 diabetes [4], and a modest positive association between plasma uric acid concentration and the incidence of type 2 diabetes in Chinese individuals was suggested by Chien et al. [5]. As is known, both insulin resistance and *β*-cell dysfunction play determinate roles in the pathogenesis of type 2 diabetes [6]. The correlation between sUA and insulin resistance has been verified by several researches years ago [7, 8], and insulin resistance is thought to be the principal underlying pathophysiologic abnormality connecting hyperuricemia and components of MetS. However, most previous studies were performed on nondiabetic subjects. Furthermore, few studies focused on the relationship between sUA level and islet *β*-cell dysfunction, and whether there is a corresponding change of sUA level with *β*-cell function deterioration in type 2 diabetes is unknown. In this cross-sectional study, we investigated the association between sUA and *β*-cell function as well as insulin sensitivity in patients with type 2 diabetes, and we further elucidate the time-dependent changes of insulin secretion ability in different gender and uric acid level groups.

## 2. Materials and Methods

### 2.1. Study Subjects

One thousand and twenty-one patients with type 2 diabetes who received treatment in the First Affiliated Hospital of Nanjing Medical University between 2008 and 2011 were enrolled in this study. Type 2 diabetes mellitus was diagnosed according to the criteria of the American Diabetes Association [9]. The maximum duration of diabetes was 35 years. The average age of all patients was 56.86 ± 12.34 (mean ± SD) years old. Meanwhile, body mass index (BMI) and sUA were 25.10 ± 3.50 kg/m^2^ and 299.08 ± 88.96 *μ*mol/L, respectively. Patients with severe pancreatic disease, liver disease, and renal disease and those who suffered recent diabetic ketoacidosis and hyperosmotic nonketotic diabetic coma were excluded. Verbal informed consent was obtained from all participants. The study was approved by the ethics committee of the First Affiliated Hospital of Nanjing Medical University.

### 2.2. Measurements

In all subjects, the height, weight, systolic blood pressure (SBP), and diastolic blood pressure (DBP) were measured and recorded. History of hypertension, family history of diabetes (FHD), and the years from diagnosis of type 2 diabetes were inquired. If one of parents, siblings, or grandparents had been diagnosed with diabetes, patient was defined as having FHD. All patients stopped using antidiabetic medicine at least one day before the blood samples are taken. After 10–12 hours overnight fasting, venous blood samples were collected to measure uric acid, glycated haemoglobin (HbA1c), triglyceride (TG), total cholesterol (TC), high-density lipoprotein cholesterol (HDL), low-density lipoprotein cholesterol (LDL), alanine aminotransferase (ALT), fasting plasma glucose (G0), and fasting serum insulin (I0). Then the 75 g oral glucose tolerance test (OGTT) and insulin secretion test were performed, and venous blood samples were obtained at 30 and 120 minutes after glucose load for measuring the plasma glucose (G30, G120) and serum insulin (I30, I120).

Plasma glucose concentrations were measured using the hexokinase method (OLYMPUS AU5400). HbA1c and serum insulin were measured by high performance liquid chromatography (Bio-Rad D10) and radioimmunoassay (Iodine [^125^I] Insulin Radioimmunoassay Kit, Beijing North Institute of Biological Technology), respectively. ALT and serum lipid profiles, including TG, HDL, and LDL, were determined with an automatic biochemical analyzer (HITACHI 7020).

### 2.3. Calculations

BMI was calculated through dividing weight (kg) by square of height (m). To evaluate the insulin secretion, InsAUC30/GluAUC30 (INSR30) was calculated as a surrogate index for the early phase insulin secretion and InsAUC120/GluAUC120 (INSR120) as a surrogate index for total insulin secretion, where InsAUC30 and GluAUC30 are the area under insulin (mIU/L) and glucose (mmol/L) curves during 0 to 30 min of the OGTT and InsAUC120 and GluAUC120 are the area under insulin and glucose curves during 0 to 120 min, respectively [10]. Matsuda insulin sensitivity index (Matsuda ISI, calculated as $10000/\sqrt{\left(\text{G}0\times \text{I}0)\times (\overset{-}{\text{G}}\times \overset{-}{\text{I}}\right)}$, where $\overset{-}{\text{G}}$ and $\overset{-}{\text{I}}$ are the average levels of plasma glucose (mg/dL) and insulin (mIU/L) during OGTT) was chosen to evaluate insulin sensitivity [11]. The homeostasis model assessment of insulin resistance (HOMA-IR) index was calculated as follows: insulin (mIU/L) × glucose (mmol/L)/22.5 [12]. Glucose disposition indices (disposition index 30 = DI30 = Matsuda ISI × INSR30, disposition index 120 = DI120 = Matsuda ISI × INSR120) [10, 13] were used to assess *β*-cell function, combining both insulin secretion and insulin sensitivity.

### 2.4. Statistical Analysis

Student's* t*-test and the chi-square test were used to analyze group differences. The correlativity between sUA and *β*-cell function was analyzed by Pearson's correlation. The multiple linear regression analysis was applied to test the associations between *β*-cell function and sUA after adjustment for several covariates. Abnormally distributed continuous variables, including HbA1c, TG, and ALT, as well as all the indices for insulin sensitivity and *β*-cell function, were log-transformed to yield an approximately normal distribution before statistical analysis. *P* value < 0.05 (two-tailed) was considered statistically significant. All statistical analyses were conducted with the Statistical Package for Social Science for Windows (SPSS, version 13.0).

## 3. Results

The characteristic of the study patients was shown in Table 1. The patients were divided into two groups according to the median sUA levels of females and males, respectively (LUA: low serum uric acid, which was under the median sUA level; HUA: high serum uric acid, which was above the median sUA level). Patients with HUA had higher levels of BMI, TG, and ALT and greater ratio of hypertension in both genders. In contrast, the HbA1c and HDL were lower in HUA groups than in LUA ones. In male, female, and total groups, HUA patients had greater HOMA-IR and smaller Matsuda ISI than LUA subjects. Consistent with insulin resistance, patients with HUA had greater insulin secretion indices, that is, INSR30 and INSR120, than LUA in all groups. Interestingly, we found that in men and total groups, patients with HUA had greater disposition indices (both DI30 and DI120) rather than in women group. Statistically significant correlations were found between sUA level and all the indices of *β*-cell function and insulin sensitivity either totally or after being stratified by gender (shown in Table 2).

To exclude confounding factors which may influence *β*-cell function, multiple linear regression was carried out using all clinical parameters which were significantly correlated with *β*-cell function indices (data of correlation was not shown) as independent variables and indices of *β*-cell function as dependent variables. The results of multiple linear regression analysis are shown in Table 3. The sUA was independently associated with INSR30 and INSR120 (*P* value of both partial regression coefficients was less than 0.001) after adjustment for sex, hypertension, years from diagnosis, BMI, HbA1c, TG, HDL, ALT, and HOMA-IR. In the regression model of DI30 and DI120, the relationship between sUA and disposition indices was statistically significant (*P* value of both partial regression coefficients was less than 0.001) after adjustment for years from diagnosis, SBP, DBP, HbA1c, TC, LDL, and HOMA-IR. After grouping patients by sex, the regression models of *β*-cell function were shown in Table 4. Serum uric acid remained significantly associated with insulin secretion indices (INSR30 and INSR120) and disposition indices (DI30 and DI120) after adjusting for potential confounding factors in both female and male groups (all *P* value of partial regression coefficients was less than 0.05).

**Figure 1:**
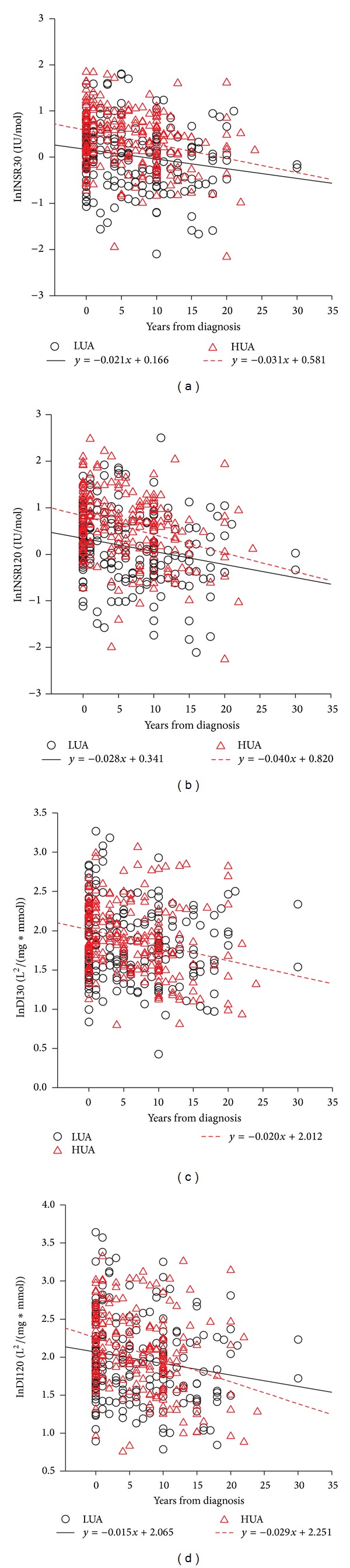
Scattered plots of simple linear regression between *β*-cell function and years from diagnosis in females. LUA, sUA levels under median of females; HUA, sUA levels above median of females. All *β*-cell function indices (INSR30, INSR30, DI30, and DI120) were log-transformed. Only significant regression lines and formulas are shown. All regression coefficients were negative. The decreases of INSR30, INSR120, and DI120 per year in the HUA group were greater than that in LUA group.

**Figure 2:**
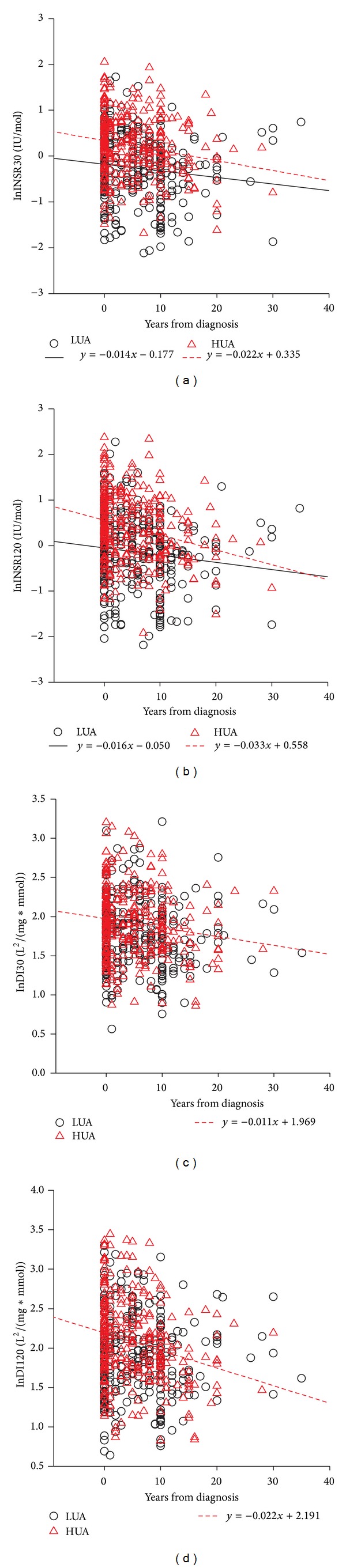
Scattered plots of simple linear regression between *β*-cell function and years from diagnosis in males. LUA, sUA levels under median of men; HUA, sUA levels above median of males. All *β*-cell function indices (INSR30, INSR30, DI30, and DI120) were log-transformed. Only significant regression lines and formulas are shown. All regression coefficients were negative. The decreases of INSR30 and INSR120 per year in the HUA group were greater than that in LUA group.

We also investigated the differences of *β*-cell function changes along with disease duration between low and high sUA levels. In the scattered plots displayed in Figures 1 and 2, in both females and males, INSR30 and INSR120 decreased along with disease duration in both HUA and LUA group (all regression coefficients were negative, *P* < 0.05). Interestingly, although INSR30 and INSR120 were higher in HUA group at the early stage of diabetes, they superficially decreased more rapidly along with disease duration than in LUA group and finally dropped to almost the same level as the LUA group. However, the difference of slop of the lines was not statistically significant after adjusting for confounding factors including hypertension, BMI, HbA1c, TG, HDL, ALT, and HOMA-IR. For disposition indices, in HUA group, DI30 and DI120 significantly dropped as disease duration increased in both females and males. However, in LUA group, no significant regression was found between disposition indices and disease duration in both genders, except for DI120 in females.

## 4. Discussion

The failure of pancreatic *β*-cell function plays an important role in the pathogenesis of type 2 diabetes. Previous studies have shown that impaired insulin secretion is the key in the conversion from normal glucose tolerance (NGT) to impaired glucose tolerance (IGT) and diabetes [14, 15], and the deterioration of *β*-cell function does not stop after diagnosis. The United Kingdom Prospective Diabetes Study (UKPDS) showed that *β*-cell function, assessed by homeostasis model assessment (HOMA), decreased approximately by 25% in the first 5 years of diabetes [16]. Several factors, including hyperglycemia, dyslipidemia, cytokines secreted by adipocytes, and immune response, have been proposed as reasons for pancreatic *β*-cell function deterioration [17–19]. In addition, our study found the close relationship between sUA and insulin secretion ability as well as glucose disposition indices, which was rarely concerned in previous studies.

Concerning the relationship between sUA level and two critical sides in the pathogenesis of type 2 diabetes, that is, insulin resistance and *β*-cell dysfunction, the interrelationship between sUA and insulin resistance was revealed by several studies years ago [7, 8]. Our present study verified this relationship again by calculating the insulin sensitivity by either Matsuda ISI or HOMA-IR. Elevated sUA level usually accompanies insulin resistance. Higher insulin levels can reduce renal excretion of urate and enhance renal urate reabsorption with increased renal tubular reabsorption of sodium [7, 20, 21]. In addition, increased purine biosynthesis and turnover, with its attendant increase in sUA, link high sUA to insulin resistance and/or hyperinsulinaemia by increased activity of the hexose monophosphate shunt [22]. On the other hand, sUA not only may be a consequence of insulin resistance but also may actually promote or worsen insulin resistance. Specifically, a recent study showed that sUA plays an important role in the pathogenesis of MetS, possibly due to its ability to inhibit endothelial function. In detail, sUA has been shown to inhibit nitric oxide (NO) bioavailability and reduce NO concentration which is required in insulin stimulated glucose uptake [23, 24]. Consequently, higher sUA always keeps with more severe insulin resistance, that is, greater insulin demand of our organs.

Few previous researches investigated the interaction between *β*-cell function and sUA. In present study, we reported the correlation between sUA level and indices reflecting islet insulin secretion ability. Subjects with higher levels of sUA had higher insulin secretion, including the early phase (INSR30) and total (INSR120) insulin secretion, and after adjustment for variables associated with insulin resistance including BMI, TG, ALT, and HOMA-IR, sUA is still independently associated with insulin secretion. The reason why patients with type 2 diabetes of HUA group can secrete more insulin at the early stage was temporarily unknown. Insulin resistance may be one of the suppositional reasons, and the increased insulin secretion may be considered as a compensatory response to overcome the insulin resistance. Although subjects with higher sUA secrete more insulin, it does not mean that high sUA is beneficial to *β*-cell function. The insulin secretion ability in those seems to drop more rapidly than those having lower sUA as type 2 diabetes duration extends. At last, when the disease duration is long enough, the difference of both INSR30 and INSR120 between HUA and LUA groups diminishes. A recent study provided evidence that sUA has a direct negative effect on *β*-cell function, which could cause *β*-cell death and dysfunction by activation of the NF-*κ*B and iNOS-NO signal axis. This may partly explain the reason why insulin secretion ability seems to drop more rapidly in HUA group [25]. Nevertheless, the profound physiology changes connecting uric acid metabolism and insulin secretion is still worth further investigation.

Disposition indices which reflect the real glucose metabolism give attention to both insulin sensitivity and insulin secretion after glucose load. In our research, the sUA always kept positive association with disposition indices in total patients and after having been divided into gender groups. This finding is consistent with the result also got in our study that those subjects with higher sUA got statistically lower HbA1c level. All these suggest that high sUA level is associated with better glucose utilization. A mechanism underlying the relationship between glucose utilization and sUA levels may be due to the uricosuric effect of glycosuria, which means hyperglycemia facilitates uric acid excretion when the blood glucose level is above 10 mmol/L [26]. By glycosuria, the sUA concentration of patients with high blood glucose levels may be low; however high blood glucose is harmful to islet *β*-cells. Furthermore, some studies demonstrated high sUA levels are associated with increased generation of free radicals and oxidative stress [27], which has various adverse effects on *β*-cell function [28, 29]. This could be one possible reason for the faster decline of insulin secretion function in high sUA patients. However, other studies have suggested that sUA is an effective antioxidant [30, 31] and elevated sUA levels may reflect a compensatory mechanism contributing to the increased oxidative stress associated with the MetS. Collectively, the exact role of sUA in oxidation is still controversial, and further research is required.

We are very cautious about making conclusion in the close relation between sUA and *β*-cell insulin secretion function as well as glucose disposition because of two points: firstly, this is just a cross-sectional study rather than longitudinal study; secondly, subjects in our study received a wide diversity of hypoglycemic therapy which might influence the nature change of the islet function.

However, from this study, an independent positive association between sUA and *β*-cell function is confirmed, suggesting a potential close relation and interaction between uric acid and insulin secretion ability. Type 2 diabetic patients with higher sUA level have better insulin secretion but their residual *β*-cell function seems to decay more quickly.

## Figures and Tables

**Table 1:** Baseline characteristics of subjects grouped by gender and serum uric acid levels (mean ± SD or number).

	Female		Male		Total	
	LUA (*n* = 209)	HUA (*n* = 210)	LUA (*n* = 301)	HUA (*n* = 301)	LUA (*n* = 510)	HUA (*n* = 511)
sUA (*μ*mol/L)	207.61 ± 38.31	339.84 ± 71.25^#^	250.19 ± 46.47	383.06 ± 61.04^#^	232.74 ± 48.08	365.29 ± 68.73^#^
Age (yr)	57.83 ± 11.11	61.40 ± 11.42^†^	55.65 ± 11.63	54.23 ± 13.52	56.54 ± 11.45	57.18 ± 13.16
Years from diagnosis	6.19 ± 6.36	6.24 ± 5.72	5.82 ± 6.22	5.16 ± 5.72	5.97 ± 6.27	5.60 ± 5.74
Hypertension (yes/no)	85/124	146/64^#^	115/186	149/152^†^	200/310	295/216^#^
FDM (yes/no)	78/131	80/130	123/178	113/188	201/309	213/308
SBP (mmHg)	133.04 ± 16.71	135.33 ± 16.52	131.33 ± 14.95	133.00 ± 16.15	132.03 ± 15.70	133.96 ± 16.33
DBP (mmHg)	81.46 ± 9.35	81.85 ± 10.29	82.56 ± 9.83	83.94 ± 9.74	82.11 ± 9.65	83.08 ± 10.01
BMI (kg/m^2^)	24.39 ± 3.67	25.87 ± 3.62^#^	24.24 ± 3.26	25.92 ± 3.22^#^	24.29 ± 3.43	25.90 ± 3.38^#^
HbA1c (%)	9.51 ± 2.51	8.85 ± 2.39^†^	9.83 ± 2.55	9.15 ± 2.39^†^	9.70 ± 2.53	9.02 ± 2.39^#^
TC (mmol/L)	4.99 ± 1.18	5.09 ± 1.15	4.70 ± 1.13	4.74 ± 1.10	4.81 ± 1.15	4.88 ± 1.13
TG (mmol/L)	1.57 ± 1.17	1.90 ± 1.30^#^	1.57 ± 1.39	2.27 ± 2.21^#^	1.56 ± 1.30	2.11 ± 1.89^#^
HDL (mmol/L)	1.25 ± 0.32	1.17 ± 0.28^†^	1.15 ± 0.32	1.01 ± 0.23^#^	1.19 ± 0.32	1.07 ± 0.26^#^
LDL (mmol/L)	3.12 ± 0.87	3.19 ± 0.84	3.00 ± 0.79	3.03 ± 0.74	3.05 ± 0.82	3.09 ± 0.78
ALT (IU/L)	25.17 ± 18.35	28.52 ± 25.71	25.65 ± 18.95	33.65 ± 25.17^#^	25.45 ± 18.69	31.54 ± 25.49^#^
INSR30	1.31 ± 1.01	1.78 ± 1.13^#^	0.99 ± 0.76	1.56 ± 1.12^#^	1.12 ± 0.88	1.65 ± 1.12^#^
INSR120	1.59 ± 1.43	2.29 ± 1.76^#^	1.18 ± 1.08	1.94 ± 1.59^#^	1.34 ± 1.25	2.08 ± 1.66^#^
DI30	7.10 ± 3.89	7.35 ± 3.59	6.49 ± 3.20	7.47 ± 3.65^#^	6.74 ± 3.50	7.41 ± 3.62^#^
DI120	8.52 ± 5.78	9.10 ± 4.94	7.61 ± 4.35	9.23 ± 5.46^#^	7.97 ± 5.00	9.17 ± 5.24^#^
HOMA-IR	2.46 ± 1.73	3.04 ± 1.86^#^	2.02 ± 1.48	2.71 ± 1.89^#^	2.20 ± 1.59	2.84 ± 1.88^#^
Matsuda ISI	7.27 ± 4.71	5.17 ± 3.11^#^	8.99 ± 5.48	6.36 ± 3.93^#^	8.28 ± 5.24	5.87 ± 3.65^#^

Abnormally distributed continuous variables, including HbA1c, TG, ALT, INS0, INS30, INS120, INSR30, INSR120, DI30, DI120, HOMA-IR, and Matsuda ISI, were log-transformed for analysis; nontransformed values were displayed for ease of interpretation. Differences between the LUA and HUA group were estimated using unpaired Student's *t*-test (or chi-square test for classified data). ^†^ *P* < 0.01; ^#^ *P* < 0.001.

**Table 2:** Correlations between serum uric acid level and *β*-cell function as well as insulin sensitivity.

	Total	Female	Male
	*r*	*r*	*r*
INSR30	0.301^#^	0.290^#^	0.395^#^
INSR120	0.296^#^	0.275^#^	0.393^#^
DI30	0.150^#^	0.100*	0.206^#^
DI120	0.166^#^	0.115*	0.226^#^
HOMA-IR	0.192^#^	0.216^#^	0.241^#^
Matsuda ISI	−0.252^#^	−0.261^#^	−0.336^#^

All abnormally distributed continuous variables were log-transformed. *P < 0.05; ^#^ *P* < 0.001.

**Table 3:** Multiple linear regression for *β*-cell function in total patients.

	INSR30		INSR120		DI30		DI120	
	*β*	S-*β*	*β*	S-*β*	*β*	S-*β*	*β*	S-*β*
Sex (0 = female, 1 = male)	−0.201	−0.136^#^	−0.229	−0.139^#^	—	—	—	—
Hypertension (yes = 1, no = 0)	−0.026	−0.018	−0.018	−0.011	—	—	—	—
Years from diagnosis	−0.020	−0.165^#^	−0.029	−0.218^#^	−0.016	−0.217^#^	−0.026	−0.287^#^
BMI (kg^2^/cm)	0.021	0.005^#^	0.018	0.076^†^	—	—	—	—
SBP (mmHg)	—	—	—	—	0.002	0.055	0.001	0.021
DBP (mmHg)	—	—	—	—	−0.005	−0.099^†^	−0.006	−0.111^#^
HbA1c (%)	−0.116	−0.400^#^	−0.156	−0.479^#^	−0.075	−0.414^#^	−0.114	−0.527^#^
sUA (*μ*mol/L)	0.001	0.147^#^	0.001	0.146^#^	0.001	0.154^#^	0.001	0.156^#^
TG (mmol/L)	−0.006	−0.005	0.015	0.012	—	—	—	—
TC (mmol/L)	—	—	—	—	−0.011	−0.029	−0.019	−0.040
HDL (mmol/L)	−0.077	−0.032	−0.098	−0.036	—	—	—	—
LDL (mmol/L)	—	—	—	—	−0.007	−0.012	0.009	0.014
ALT (IU/L)	0.091	0.076^†^	0.103	0.077^†^	—	—	—	—
HOMA-IR	0.497	0.455^#^	0.426	0.349^#^	−0.222	−0.326^#^	−0.279	−0.343^#^

*Β*: partial regression coefficient; S-*β*: standard partial regression coefficient. All abnormally distributed continuous variables were log-transformed. Only parameters which were independently associated with *β*-cell function indices after multiple stepwise regression are shown. ^†^ *P* < 0.01; ^#^ *P* < 0.001.

**Table 4:** Multiple linear regression for *β*-cell function after grouping by gender.

	INSR30		INSR120		DI30		DI120	
	*β*	S-*β*	*β*	S-*β*	*β*	S-*β*	*β*	S-*β*
Female								
Hypertension (yes = 1, no = 0)	−0.016	−0.012	−0.023	−0.015	—	—	—	—
Years from diagnosis	−0.024	−0.216^#^	−0.034	−0.268^#^	−0.020	−0.256^#^	−0.030	−0.331^#^
BMI (kg^2^/cm)	0.015	0.082*	0.010	0.050	—	—	—	—
SBP (mmHg)	—	—	—	—	0.001	0.051	0.001	0.008
DBP (mmHg)	—	—	—	—	−0.006	−0.125*	−0.006	−0.111*
HbA1c (%)	−0.114	−0.409^#^	−0.155	−0.490^#^	−0.069	−0.366^#^	−0.109	−0.491^#^
sUA (*μ*mol/L)	0.001	0.115^†^	0.001	0.107^†^	0.001	0.143^†^	0.001	0.143^#^
TG (mmol/L)	0.021	0.017	0.046	0.032	—	—	—	—
TC (mmol/L)	—	—	—	—	−0.052	−0.130	−0.074	−0.159
HDL (mmol/L)	−0.065	−0.029	−0.131	−0.052	—	—	—	—
LDL (mmol/L)	—	—	—	—	0.039	0.070	0.063	0.098
ALT (IU/L)	0.106	0.096*	0.117	0.094*	—	—	—	—
HOMA-IR	0.456	0.435^#^	0.412	0.345^#^	−0.275	−0.384^#^	−0.309	−0.367^#^
Male								
Hypertension (yes = 1, no = 0)	−0.024	−0.016	−0.006	−0.004	—	—	—	—
Years from diagnosis	−0.017	−0.134^#^	−0.026	−0.186^#^	−0.014	−0.187^#^	−0.023	−0.258^#^
BMI (kg^2^/cm)	0.026	0.116^#^	0.024	0.100^†^	—	—	—	—
SBP (mmHg)	—	—	—	—	0.001	0.034	0.001	0.021
DBP (mmHg)	—	—	—	—	−0.003	−0.056	−0.005	−0.086*
HbA1c (%)	−0.116	−0.395^#^	−0.154	−0.474^#^	−0.075	−0.423^#^	−0.112	−0.526^#^
sUA (*μ*mol/L)	0.001	0.163^#^	0.002	0.169^#^	0.001	0.201^#^	0.001	0.212^#^
TG (mmol/L)	−0.026	−0.023	0.001	0.001	—	—	—	—
TC (mmol/L)	—	—	—	—	0.004	0.033	0.002	0.003
HDL (mmol/L)	−0.088	−0.034	−0.064	−0.022	—	—	—	—
LDL (mmol/L)	—	—	—	—	−0.036	−0.063	−0.021	−0.030
ALT (IU/L)	0.075	0.060*	0.083	0.060*	—	—	—	—
HOMA-IR	0.522	0.469^#^	0.430	0.350^#^	−0.202	−0.304^#^	−0.283	−0.353^#^

*Β*: partial regression coefficient; S-*β*: standard partial regression coefficient. All abnormally distributed continuous variables were log-transformed. Only parameters which were independently associated with *β*-cell function indices after multiple stepwise regression are shown. *P < 0.05; ^†^ *P* < 0.01; ^#^ *P* < 0.001.
